# Long-Term Consumption of a Sugar-Sweetened Soft Drink in Combination with a Western-Type Diet Is Associated with Morphological and Molecular Changes of Taste Markers Independent of Body Weight Development in Mice

**DOI:** 10.3390/nu14030594

**Published:** 2022-01-29

**Authors:** Barbara Lieder, Jozef Čonka, Agnes T. Reiner, Victoria Zabel, Dominik Ameur, Mark M. Somoza, Katarína Šebeková, Peter Celec, Veronika Somoza

**Affiliations:** 1 Department of Physiological Chemistry, Faculty of Chemistry, University of Vienna, 1090 Vienna, Austria; agnes.mistlberger-reiner@univie.ac.at (A.T.R.); victoria.zabel@hotmail.com (V.Z.); veronika.somoza@univie.ac.at (V.S.); 2 Institute of Molecular Biomedicine, Faculty of Medicine, Comenius University, 81101 Bratislava, Slovakia; dodis.conka@gmail.com (J.Č.); kata.sebekova@gmail.com (K.Š.); petercelec@gmail.com (P.C.); 3 Department of Inorganic Chemistry, Faculty of Chemistry, University of Vienna, 1090 Vienna, Austria; dominik.ameur@univie.ac.at (D.A.); mark.somoza@univie.ac.at (M.M.S.); 4 Leibniz Institute for Food Systems Biology at the Technical University of Munich, 85345 Freising, Germany; 5 Food Chemistry and Molecular Sensory Science, Technical University of Munich, 85345 Freising, Germany; 6 Nutritional Systems Biology, School of Life Sciences, Technical University of Munich, 85345 Freising, Germany

**Keywords:** taste dysfunction, diet, mice, sugar-sweetened beverage, Western diet

## Abstract

We investigated whether the long-term intake of a typical sugar-sweetened soft drink (sugar-sweetened beverage, SSB) alters markers for taste function when combined with a standard diet (chow) or a model chow mimicking a Western diet (WD). Adult male CD1 mice had ad libitum access to tap water or SSB in combination with either the chow or the WD for 24 weeks. Energy intake from fluid and food was monitored three times a week. Cardiometabolic markers (body weight and composition, waist circumference, glucose and lipid profile, and blood pressure) were analyzed at the end of the intervention, as was the number and size of the fungiform papillae as well as mRNA levels of genes associated with the different cell types of taste buds and taste receptors in the circumvallate papillae using a cDNA microarray and qPCR. Although the overall energy intake was higher in the WD groups, there was no difference in body weight or other cardiometabolic markers between the SSB and water groups. The chemosensory surface from the fungiform papillae was reduced by 36 ± 19% (*p* < 0.05) in the WD group after SSB compared to water intake. In conclusion, the consumption of the SSB reduced the chemosensory surface of the fungiform papillae of CD1 mice when applied in combination with a WD independent of body weight. The data suggest synergistic effects of a high sugar-high fat diet on taste dysfunction, which could further influence food intake and promote a vicious cycle of overeating and taste dysfunction.

## 1. Introduction

The prevalence of overweight and obesity has been constantly increasing worldwide during the past decade. One major factor that contributes to the multifaceted origin of the obesity epidemic is the overconsumption of highly palatable, energy-dense foods with high contents of fat, salt, and carbohydrates such as sugars [1,2]. The intake of energy-dense, ultra-processed food with little nutritive value, providing 58% of the energy intake and 89% of added sugars in an American diet, is associated with excess body weight [3]. Studies show that foods with a high fat or sugar content are preferred by obese people [4,5,6], which further increases weight gain, and with that, obesity. Food intake is regulated by multiple factors that can be divided into physiological factors (e.g., metabolic status), cognitive-affective factors (e.g., mood, stress), and socioeconomic factors (e.g., culture, food availability) [7]. Still, one of the most important drivers of food choice remains gustation, the sense of taste, as it is evolutionary helping to distinguish between nutritive and potentially harmful foods [8]. However, although useful from an evolutionary perspective, the preferred taste of fat and sugar also determines the palatability of foods, which may contribute to overeating. In general, taste perception is mediated via taste buds, which represent the actual taste organs. Taste buds are located in the fungiform papillae, which are distributed at the dorsal surface of the tongue, the larger circumvallate papillae at the posterior tongue, and the foliate papillae on the lateral sides of the tongue. Each taste bud contains around 50–100 epithelial cells with different specialized functions. Taste cell type II and III, and probably also type I cells, carry receptors that mediate sweet, bitter, umami, salty, and sour taste. In addition, taste buds also contain stem cells and basal cells, which give rise to new taste cells (summarized by Roper and Chaudhari, [9]).

Recent studies indicated that taste function in obese individuals is disturbed, and that obesity directly impairs taste buds [10,11], which has been mechanistically addressed using mouse models. In C57Bl/6 mice, fewer circumvallate taste buds [10], and a lower number of fungiform papillae, along with a reduced expression of type I, II, and III taste cell markers [11] after eight weeks of an obesogenic diet has been reported. Moreover, in C57Bl/6 mice on a high-fat diet for 10 weeks, obesity was accompanied by a reduced number of taste cells, and a reduced responsiveness to sweet stimuli was shown using calcium imaging [12]. Likewise, in high-fat diet-induced obese rats, a lower expression of the sweet and umami taste receptor subunit Tas1r3 was detected, accompanied by a reduced preference for a sweet-tasting saccharin solution at lower concentrations, suggesting a reduced sensitivity to sweet taste [13]. In addition, one recent study by Ahart et al. [14] addressed the question as to whether the changes to the taste organs are due to the increased body weight or rather the exposure to the high-fat diet. In this study, C57Bl/6 mice received either a high-fat diet or standard chow for six to eight weeks in the absence or presence of captopril, which prevents weight gain. The results revealed that exposure to a high-fat diet without body weight gain led to a decreased expression of the taste signaling molecules α-gustducin and phospholipase b2 [14]. Moreover, a previous study in *Drosophila melanogaster* demonstrated that a high-sugar diet, but not the non-caloric sweetener sucralose, decreased the nerve responses to sweet stimuli, accompanied by extended, larger meals and the development of obesity. Restoring the excitability of the sweet-sensing neurons by genetic modification prevented overconsumption, and thus diet-induced obesity [15]. This study highlights that at least in fruit flies, obesity is not necessary for a dysfunction in sweet taste, and the dietary intervention with sugar was sufficient to induce taste dysfunction. However, studies investigating the effect of the long-term intake of high amounts of sugar in mice on taste markers are lacking, and potentially additive or synergistic effects of sugar consumption on a Western-type diet have not been addressed so far. Thus, this study investigated for the first time whether the long-term intake of a caffeinated sugar-sweetened soft drink alters markers for the taste function in mice when applied in combination with a regular chow (chow) or a high-fat Western-type diet (WD) in relation to a broad spectrum of biomarkers of the metabolic syndrome. We hypothesized that the metabolic and taste changes associated with high sugar intake are higher when applied in combination with a typical WD high in fats and carbohydrates. To explore this hypothesis, CD1 mice received either a chow or WD in combination with water or a sugar-sweetened beverage (SSB) for 24 weeks. At the end of the experiment, biomarkers of the metabolic syndrome as well as morphological and molecular taste markers were investigated.

## 2. Materials and Methods

### 2.1. Study Design

All experimental practices were carried out in compliance with EU Guidelines for Scientific Experimentation on Animals and were approved by the ethics committee of the Institute of Pathophysiology, Comenius University, Bratislava, Slovakia on 9 July 2015, and the State Veterinary and Food Administration of the Slovak Republic on 7 December 2015 under protocol code 3939/15-221. 

Nine-week-old CD1 mice were purchased from Velaz, Prague, Czech Republic, and housed in conventional cages at a room temperature between 21–24 °C and 55–65% humidity with a 12 h-light-dark cycle and ad libitum access to food and drink. After a week-long quarantine when they were administered a standard chow, the animals were divided into four groups with a similar body weight. Two of the groups received chow and two groups received conventional cheeseburgers as a typical Western-type diet (WD), one subgroup per diet receiving tap water and the other group a regular, commercially available caffeinated sugar sweetened soft drink as the prototypical SSB. The duration of the intervention was 24 weeks. 

The cheeseburgers were obtained from a local restaurant in Bratislava (Slovakia) and ground with a meat grinder, carefully mixed, and formed to appropriate, homogeneous chunks of approximately 2 × 5 cm. The WD chunks were stored at −20 °C for a maximum of three weeks until they were offered to the mice. The composition of the cheeseburgers was 39% fat, 41% carbohydrates, and 19% proteins and provided an energy content of 11 MJ/kg. The standard chow maintenance pellet food (V1534-000) (Sniff, Soest, Germany) consisted of 9% fat, 58% carbohydrates, and 33% protein, providing 12.9 MJ/kg of metabolizable energy. The SSB contained 11.2% glucose-fructose syrup as a sweetener and was obtained from a local supermarket in the Bratislava region (Slovakia). It was decarbonated by stirring to prevent leakage from the feeding bottle before providing it to the mice. The mice were euthanized by over dose of isoflurane (Priamal Healthcare, London, UK), blood was collected from the retroorbital sinus for further analysis, and the brown adipose tissue (BAT) located between the shoulder blades was harvested. 

### 2.2. Body Weight and Composition 

Body weight and body composition were recorded at the end of the experiment. Body composition was evaluated by non-invasive dual-energy X-ray absorptiometry (DEXA) using the LUNAR Prodigy Advance device (GE Medical Systems, Madison, WI, USA), and each mouse was scanned five times. Based on the attenuation of the two energy levels, EncoreTM 2011 software for small animals was used to obtain the content of fat and lean tissue. The waist circumference was measured at the perimeter of the body at the level of the belly button using a measuring tape. 

### 2.3. Insulin Sensitivity 

Blood glucose concentrations were measured using a standard glucose meter (FreeStyle Precision Neo Meter, Abbott, Chicago, IL, USA). Insulin concentration was determined by using a commercially available ELISA (Insulin ELISA, Mercodia, Uppsala, Sweden). 

The quantitative insulin sensitivity check index (QUICKI) was used as a species-independent marker for glucose tolerance [16] and it was calculated using the following formula [17]:

QUICKI = 1/(log(fasting insulin IU/L) + log(fasting glucose mmol/L)).

An oral glucose tolerance test (oGTT) was performed by administration of 2 g/kg of glucose in 500 µL of water by gavage after three hours of fasting. Blood samples were taken from the tip of the tail at time points 0, 15, 30, 60, 90, and 120 min to analyze glucose concentrations after the glucose challenge. The area under the glucose curve (AUC) was calculated.

### 2.4. Blood Lipid Analysis

In the blood samples obtained at the end of the experiment, total cholesterol, high density lipoprotein (HDL-C), low-density lipoprotein (LDL-C) cholesterol, and triacylglycerols (TAG) levels were determined using the standard laboratory methods (Biolis 24i Premium instrument, Tokyo, Boeki, Japan). 

### 2.5. Body Temperature

For the determination of the body temperature, CD1 mice were fixed dorsally on a stable platform during the measurement and another researcher took an image with the thermal imaging camera (FLIR-E64501) directly from the frontal side of the mouse. Lenses of the camera were focused on the facial part of the mouse and the ocular surface temperature was captured three times from a constant distance according to the protocol of Vogel et al. [18]. The hottest temperature-labeled area with a red rectangle was recorded and the representative image was documented. Thermal images were then analyzed using FLIR Tools (FLIR Systems, Inc., Wilsonville, OR, USA) software. The average temperature of the hottest area from each measurement on the ocular facial area was considered as the body temperature of the animal.

### 2.6. Blood Pressure & Heart Rate 

Blood pressure and heart rate were assessed during the last week of the trial using the noninvasive tail cuff volume-pressure (VPR) method (CODA™, Kent Scientific Corporation, Torrington, CT, USA). First, the mice were pre-warmed at 37 °C on a heating plate before being placed in the restrainer. A tail and a VPR cuff were pushed up near the base of the tail. Systolic, diastolic blood pressure, and heart rate were measured a total of 15 times. The first five measurements were used to habituate the animal to the measurement, and the remaining 10 measurements were used to determine an average value for systolic, diastolic blood pressure, and heart rate. 

### 2.7. Cardiometabolic Risk Estimation

For each animal, *z*-score of systolic blood pressure (SBP), waist circumference, fasting glucose, TAG, and HDL-C concentration was calculated. Cardiometabolic risk was estimated via the calculation of continuous metabolic syndrome score (cMSS), as the SBP-*z*-score + waist circumference-*z*-score + glycemia-*z*-score + TAG-*z*-score + HDL-C-*z*-score (inverted). The higher the cMSS, the higher the cardiometabolic risk.

### 2.8. Number and Size of Fungiform Papillae

The method of tongue surface observation was adopted according to the description by Mistretta et al. [19], using intact, stained tongues. In detail, the number and size of the fungiform papillae was analyzed at the anterior part in front of the intermolar eminence of each tongue after staining the tongues with 0.5% (*w*/*v*) of methylene blue in water for one minute. The excess dye was removed by a brief washing step in PBS before the tongue was placed between two glass slides for further analysis. The fungiform papillae stains more lightly than the non-gustatory filiform papillae and can thus be easily identified. The fungiform papillae were counted under a dissecting microscope (Eschenbach Optik GmbH, Nuremberg, Germany) in duplicates by the same investigator. 

The size of the papillae was calculated using the software Image J (Version 1.8.0._112, LOCI, University of Wisconsin, Madison, WI, USA) based on an average of 16 fungiform papillae from a mean of four pictures from different regions of the anterior tongue taken at 5× magnification on the Axio Lab A1 microscope (Carl Zeiss, Jena, Germany) coupled to an Axiocam ERc 5s camera (Carl Zeiss, Jena, Germany) and Zen 2.3 Lite Software (Carl Zeiss, Jena, Germany). 

### 2.9. Gene Expression Analysis of Taste Markers

The circumvallate papillae (CV) was chosen for the gene expression analysis since it bears a high density of taste buds containing the highest numerical density of type II and III taste cells [20,21]. The posterior part of the tongue containing the CV was separated from the anterior part and incubated for at least 16 h at −20 °C in pre-cooled RNAlater-ICE (Invitrogen, ThermoFisher Scientific, Vienna, Austria). Total RNA was isolated from the extracted CV using the Monarch Total RNA Miniprep Kit (New England Biolabs GmbH, Frankfurt am Main, Germany) following the manufacturer’s protocol for tissue samples. A DNAase I treatment was performed to remove genomic DNA during the procedure. The quality of the purified RNA was checked photometrically by analyzing the 260/280 and 260/230 nm ratios using a NanoQuant Plate on an Infinite M 200 plate reader (Tecan, Menningen, Switzerland) and the integrity was controlled by agarose gel electrophoresis. The obtained RNA samples from each mouse were split in two parts, one half was further used for transcriptome analysis using a customized microarray and the other half was used for qRT-PCR analysis. 

### 2.10. Microarray Analysis

A customized microarray containing 60-mer oligonucleotide probes to analyze genes for chemosignaling was synthesized using the technique of maskless synthesis as described previously [22]. The probes for the selected taste markers were based on the SurePrint G3 Mouse Gene Expression v2 Microarray (Agilent, Santa Clara, CA, USA). 

RNA samples were pooled by group and the RNA content of each pool was adjusted to the RNA amount of the pool with the lowest content, yielding a total of 1.7 µg of RNA per group for reverse transcription. The samples were labeled according to the protocol described by [23]. In brief, samples were labeled using Cy3-labled random nonamers during reverse transcription reaction using SuperScript III. The reaction was stopped and the remaining RNA removed by the addition of 1 volume of 200 mM NaOH/20 mM EDTA solution and incubated for 10 min at 65 °C. The samples were neutralized by the addition of 1 volume of 1 M HEPES and the samples purified using Qiaquick PCR purification columns (Qiagen, Hilden, Germany) according to the manufacturer’s protocol. The labeled cDNA was hybridized to the microarray at 42 °C for 22 h. Scanning of the washed arrays was accomplished on a GenePix 4400 microarray scanner (Molecular devices, Sunnyvale, CA, USA). The fluorescence intensities for each probe were extracted from the scanned images using NimbleGen 2.1 software (NimbleGen, Madison, WI, USA) and normalized using the robust multichip averaging (RMA).

### 2.11. qRT PCR

The isolated RNA samples per mouse were reverse transcribed to cDNA using the LunaScript RT Supermix Kit (New England Biolabs GmbH, Frankfurt am Main, Germany). Real Time PCR analysis was carried out using the Luna Universal qPCR Mastermix (New England Biolabs GmbH, Frankfurt am Main, Germany) in triplicates on a StepOnePlus Real Time PCR device (Applied Biosystems, Thermo Fisher Scientific, Waltham, MA, USA). The respective mRNA starting concentrations were estimated based on the threshold and mean PCR efficiencies per gene using LinReg PCR (version 2018.0) [24] and potential plate effects were excluded by normalization with Factor qPCR (version 2016.0) [25]. Each gene was normalized to the geometric mean of three reference genes (*Actb*, *Hprt*, *18s*). Primers used for PCR analysis were synthesized and obtained from Sigma Aldrich Austria (Merck Life Science, Vienna, Austria) and details are provided in Supplementary Material Table S1. Primers that were not previously described were validated by sequencing the obtained PCR product (Eurofins Genomics AT GmbH, Vienna, Austria).

### 2.12. Statistical Analysis

Raw data were tested for outliers using the Rout test (Q = 5%) in Graph Pad Prism (version 8 or higher, San Diego, CA, USA). Data are shown as mean or mean fold change ± SEM. The absolute residuals were tested for homogeneity according to Levene’s test with MS Excel 2019 and normal distribution of the data sets was tested using the D’Agostino–Pearson normality test or the Kolmogorov–Smirnoff test in case of smaller data sets in Graph Pad Prism before a two-way ANOVA with Holm–Sidak post hoc, or for comparison of two groups, a Student’s *t*-test, was carried out as indicated in the figure legends. In case the assumptions of normal distribution and/or equality of variances were not met, a reciprocal transformation of the data was performed. If the criteria were still not met, a non-parametric test without the calculation of interactions (one-way ANOVA on ranks with Dunn’s multiple comparison test or the Mann–Whitney U test) was applied as indicated in the figure legends. A Pearson’s Product Moment correlation analysis was used to determine associations between the selected cardiometabolic markers or cardiometabolic markers and taste markers. Significant correlations were validated using scatterplots. Significance for all statistical analyses was assumed at *p* < 0.05 and trends are reported for *p* < 0.1.

## 3. Results

### 3.1. Energy Intake

**Figure 1:**
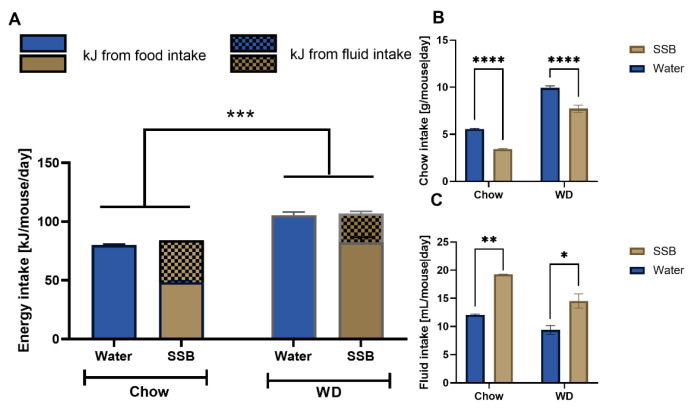
(**A**) Mean total energy intake ± SEM [kJ] from food (plain bars) and fluid intake (checkered bars) from mice receiving either a standard diet (chow) or Western-type diet (WD) with water (Water, blue bars) or a SSB (brown bars) as a drink. (**B**) Mean food intake ± SEM [g/mouse per day] and (**C**) mean fluid intake ± SEM [mL/mouse per day] from mice receiving either a standard diet (chow) or Western-type diet (WD) with water (Water, blue bars) or a sugar-sweetened beverage (SSB, brown bars) as a drink. Statistical significance was tested using two-way ANOVA with a Holm–Sidak post hoc test, expect for fluid intake, which was analyzed with an ANOVA on ranks followed by Dunn’s multiple comparison test due to a lack of equal variances, with *n* = 7–8 for the chow group, *n* = 11–12 for the WD group. Significance was assumed at *p* < 0.05 and is marked by * *p* < 0.05, ** *p* < 0.01, *** *p* < 0.001 and **** *p* < 0.0001.

The combined energy intake from fluid and solid food was on average 26% higher (*p* < 0.001) in the WD groups (115 ± 7.5 kJ/mouse per day for water group and 108 ± 3.0 kJ/mouse per day for the SSB group) compared to chow groups (80.5 ± 0.9 kJ/mouse per day for water group and 84.2 ± 1.2 kJ/mouse per day for the SSB group) but did not differ between SSB and water-fed mice in each group (Figure 1A). The SSB-fed mice consumed less chow (Figure 1B) and more fluid (Figure 1C) in both diet-groups (*p* < 0.0001). In more detail, the mice consumed on average 2.12 ± 0.22 g/ mouse per day less chow in the chow group, and 2.22 ± 0.27 g/ mouse per day less chow in the WD group when SSB was provided as beverage. In contrast, the fluid intake was higher when mice were provided SSB compared to water, there was an increase by 7.2 ± 0.7 mL/day per mouse in the chow group and by 5.1 ± 0.9 mL/day per mouse in the WD group (*p* < 0.0001), corresponding to an increase of 37% or 35%, respectively. The mean calories consumed from the soft drink were 36 kJ, or 29 kJ, respectively. Two-way ANOVA confirmed that there was no interaction between fluid intake based on the diet, meaning that the SSB intake did not depend on the type of diet. 

### 3.2. Cardiometabolic Markers

**Figure 2:**
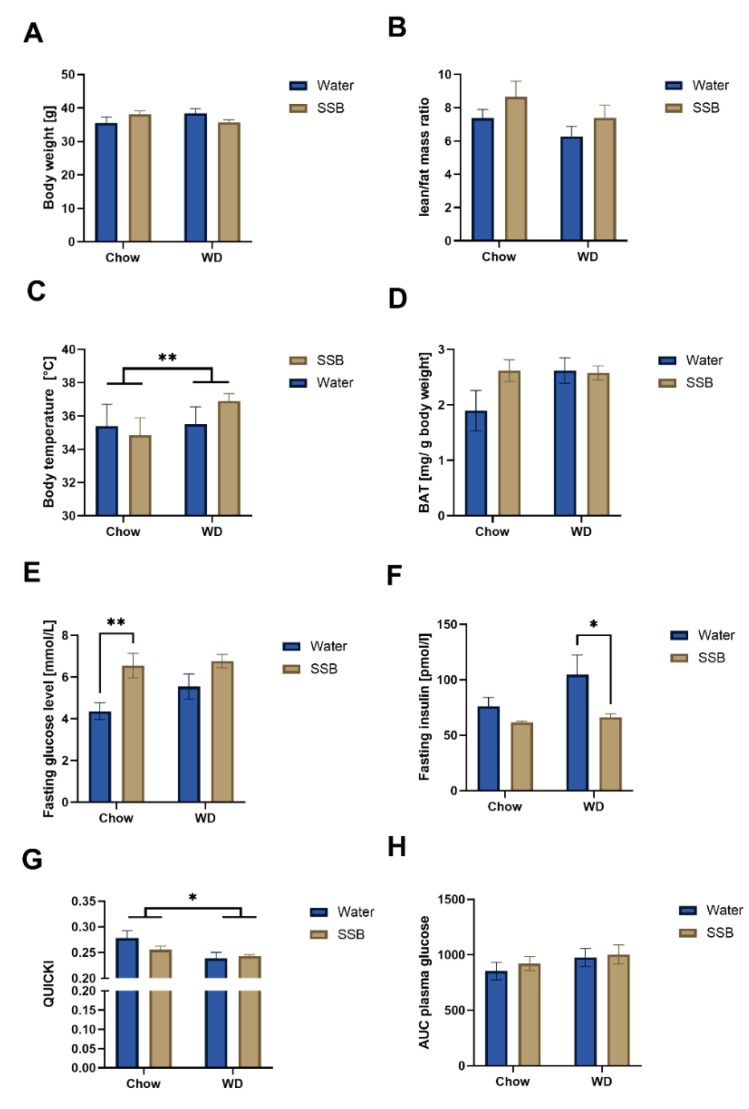
(**A**) Mean body weight (*n* = 11–12 for chow group, *n* = 7–8 for WD group), (**B**) mean lean/fat mass ratio, (*n* = 11–12 for chow group, *n* = 7–8 for WD group), (**C**) total mass of brown adipose tissue (BAT), (*n* = 9–12 for chow group, *n* = 7–8 for WD group), (**D**) mean body temperature (*n* = 11–12 for chow group, *n* = 5–8 for WD group), (**E**) mean fasting glucose level, (*n* = 11–12 for chow group, *n* = 7–8 for WD group), (**F**) mean fasting plasma insulin concentrations (*n* = 11–12 for chow group, *n* = 7–8 for WD group), (**G**) mean values for quantitative insulin sensitivity check index (QUICKI) (*n* = 9–11 for chow group, *n* = 7–8 for WD group), and (**H**) mean area under the curve (AUC) obtained from glucose concentrations during an oral glucose tolerance test (*n* = 11–12 for chow group, *n* = 7–8 for WD group). Data is shown as mean ± SEM, from mice receiving either a standard diet (chow) or Western-type diet (WD) with water (Water, blue bars) or a sugar-sweetened beverage (SSB, brown bars) as a drink for 24 weeks. Statistical significance was tested using two-way ANOVA with the Holm–Sidak post hoc test, except for quantitative insulin sensitivity check index (QUICKI), which was analyzed using the Mann–Whitney U test due to a lack of equal variances. Significance was assumed at *p* < 0.05. * *p* < 0.05, ** *p* < 0.01.

The results of the body composition analysis are displayed in Figure 2. Despite the increased energy intake in the WD group, the mean body weight did not differ between the different feeding groups (two-way ANOVA, *p* > 0.05) after 24 weeks (Figure 2A). Likewise, the mean lean/fat mass ratio, and waist circumference (data not shown) was not different between the different feeding groups (Figure 2B), albeit the body weight correlated with the fat body mass (r = 0.33, *p* < 0.05) and inversely correlated with the lean body mass (r = 0.71, *p* < 0.001). Although the amount of BAT was similar between the groups (Figure 2C), the mean body temperature was higher in mice consuming the WD (36.2 °C vs. 35.1 °C, *p* < 0.01, Figure 2D). 

In addition, two-way ANOVA analysis revealed an impact of the consumed beverage on fasting glucose concentrations (*p* < 0.01, Figure 2E). Multiple comparison showed an increased fasting glucose concentration in mice that received SSB (6.6 ± 0.6 mmol/L) in comparison to water (4.4 ± 0.4 mmol/L) in the chow group (*p* < 0.01), but not in the WD group. Both groups of mice receiving the WD had mean blood glucose concentrations of 6.5 ± 0.4 mmol/L in the SSB and 5.6 ± 0.6 mmol/L in the water group, which is higher than the chow/water group (Figure 2E). In addition, there was a direct relationship between fasting glucose concentrations and the waist circumference (r = 0.374, *p* < 0.05, Pearson’s correlation). The fasting plasma insulin concentrations were significantly higher in the WD/water group compared to the WD/SSB group (*p* < 0.05, Figure 2F). Moreover, QUICKI, as a marker for insulin sensitivity, was lower in mice fed with the WD compared to the chow (*p* < 0.05, Figure 2G). Still, there was no difference in the glucose area under the curve during the oral glucose tolerance test (oGTT) (Figure 2H). 

Plasma concentrations of TAG, total cholesterol, HDL-C, and LDL-C did not differ between the mice receiving either chow or WD with SSB or water as a drink (Supplementary Material Figure S1). However, as expected, the body fat mass correlated with LDL-C (r = 0.41, *p* < 0.05). In addition, there was neither a difference in the systolic and diastolic blood pressure, nor in the heart rate between the different feeding groups at the end of the experiment (Supplementary Material Figure S2). The mean continuous metabolic syndrome score showed no differences between the groups (Supplementary Material Figure S3).

### 3.3. Chemosensory Surface

**Figure 3:**
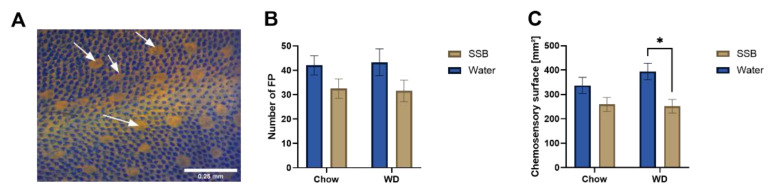
(**A**) Representative image for the analysis of the fungiform papillae after staining with methylene blue taken at 10× magnification. The light-stained fungiform papillae with the darker taste pore in the middle, exemplarily marked with arrows, can be easily distinguished from the non-gustatory filiform papillae (dark blue stained). (**B**) Mean number ± SEM of fungiform papillae at the anterior part in front of the intermolar eminence, and (**C**) mean chemosensory surface ± SEM from the fungiform papillae, calculated from the size and number of the fungiform papillae from mice receiving either a standard diet (chow) or Western-type diet (WD) with water (Water, blue bars) or a sugar-sweetened beverage (SSB, brown bars) as a drink for 24 weeks. Statistical significance was tested using two-way ANOVA with the Holm–Sidak post hoc test from *n* = 11–12 for the chow group, and *n* = 7–8 for the WD group, assumed at *p* < 0.05 and is marked by *.

The total number of fungiform papillae at the anterior part in front of the intermolar eminence was determined and the results of the morphological surface analysis are shown in Figure 3. Two-way ANOVA revealed an impact of the type of fluid on the number of fungiform papillae (*p* < 0.05), which was independent of the type of chow. Moreover, the chemosensory surface of the fungiform papillae was analyzed based on the number and area of the fungiform papillae and showed a significant reduction in mice receiving SSB compared to water in the WD group (*p* < 0.05). This effect did not reach statistical significance in the chow groups (*p* = 0.12). 

### 3.4. Gene Expression Analysis Circumvallate Papillae

**Figure 4:**
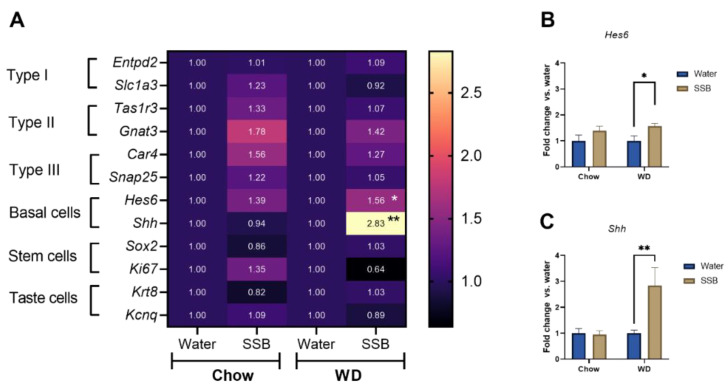
qPCR analysis of marker genes for different cell types of the taste bud in the circumvallate papillae (CV) from mice that received either a standard diet (chow) or Western-type diet (WD) with water (Water, blue bars) or a sugar-sweetened beverage (SSB, brown bars) as a drink for 24 weeks. (**A**) Heatmap representing the mean fold changes to the corresponding water control in a color code. (**B**,**C**) Gene expression analysis of *Hes6* and *Shh*. Statistical significance was tested using two-way ANOVA with the Holm–Sidak post hoc test or Student’s *t*-test, assumed at *p* < 0.05. * *p* < 0.05, ** *p* < 0.01, *n* = 9–10 for the chow group, *n* = 7–8 for the WD group.

First, two marker genes for all taste cells, cell types I-III, basal cells, and stem cells were selected based on previous studies [26] and analyzed by means of qRT PCR in the isolated circumvallate papillae. Marker genes for the taste receptor carrying type I cells (*Entpd2*, *Slc1a3*), type II cells (*Tas1r3*, *Gnat3*), and type III cells (*Car4*, *Snap25*) were not significantly different in the SSB groups compared to water, which was also confirmed by unchanged marker genes for all taste cells, *Krt8* and *Kcnq1* (Figure 4A). While the marker genes for stem cells *Sox2* and *Ki67* were not different between SSB and water groups in both diets, the marker genes for basal cells, *Hes6* and *Shh*, were significantly upregulated in mice that consumed SSB compared to water in the WD group. In more detail, the *Hes6* gene expression was increased to a fold change of 1.56 ± 0.11 (*p* < 0.05, Figure 4B), and *Shh* to 2.83 ± 0.69 (*p* < 0.01, Figure 4C). In contrast, there was no significant difference in gene expression between mice receiving SSB or water in the chow groups.

**Figure 5:**
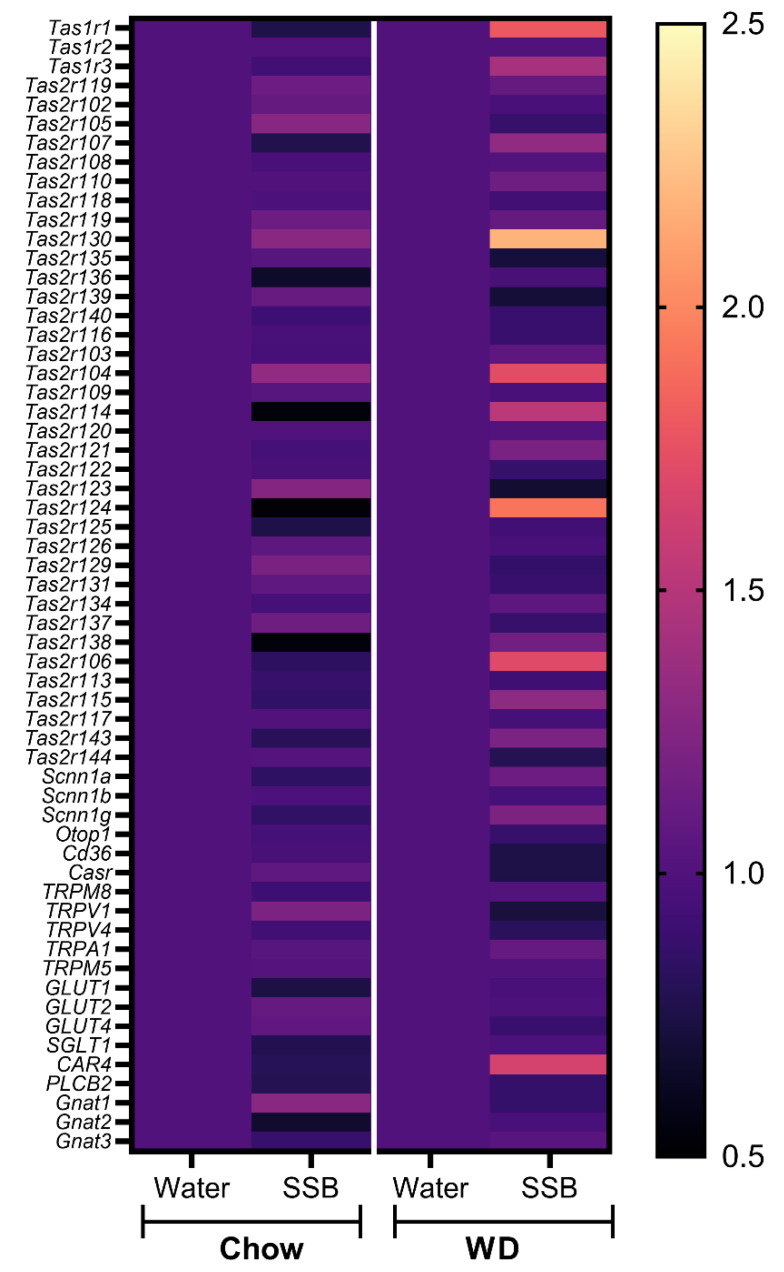
Transcriptome analysis of 59 selected genes associated with oral chemosensation, displayed as a heatmap showing mean fold changes in gene expression of the SSB-fed diet groups in relation to the respective water-fed group (=1) in the form of a color code. The gene expression was analyzed using one customized cDNA microarray per group from pooled RNA samples of the CV from mice that received either a standard diet (chow, *n* = 10–11) or Western-type diet (WD, *n* = 7–8) with water (Water) or a sugar-sweetened beverage (SSB) as a drink for 24 weeks.

Moreover, potential changes of selected genes (*n* = 59) that have been previously associated with oral chemosensation (taste receptors, TRP-channels, glucose transporters, and taste signaling) were screened with a customized cDNA-microarray and selected genes further validated by RT-qPCR. Overall, the taste-relevant genes showed a stronger tendency for upregulation after SSB consumption in the WD group in comparison to the chow group, as can be seen from the heatmap in Figure 5. The most pronounced regulation was seen for genes encoding for the bitter taste receptors Tas2r130, Tas2r104, Tas2r106, and Tas2r124 with fold changes of 2.18, 1.72, 1.70, and 1.92 in the SSB group in mice receiving a WD compared to their water-fed littermates. Furthermore, regulated genes identified using the microarray are *Car4* (fold change 1.64), *Tas1r1* (fold change 1.7), and *Tas1r3* (fold change 1.42). Based on the results obtained from the microarray, gene expression of the named bitter taste receptors, and additionally genes involved in sweet sensing pathways (*Tas1r2*, *Tas1r3*, and glucose transporter 1 *Slc2a1*) were further investigated by RT-qPCR separately for each mouse.

**Figure 6:**
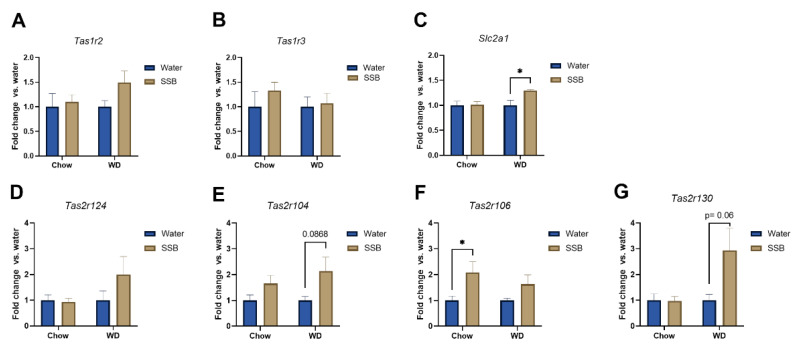
Gene expression analysis with qPCR of (**A**) *Tas1r2*, (**B**) *Tas1r3*, and (**C**) *Slc2a1*, encoding for the glucose transporter one, (**D**) *Tas2r124*, (**E**) *Tas2r104*, (**F**) *Tas2r106*, and (**G**) *Tas2r130*. RNA was obtained from the CV of mice that received either a standard diet (chow) or Western-type diet (WD) with water (Water, blue bars) or a sugar-sweetened beverage (SSB, brown bars) as a drink for 24 weeks. Mean fold changes ± SEM in gene expression of the SSB-fed diet groups is displayed in relation to the respective water-fed group (=1). Statistical significance was tested using Student’s *t*-test or Mann–Whitney U test in case of Tas2r106 and Tas2r130 and assumed at *p* < 0.05 and is marked by *, *n* = 9–10 for chow group, *n* = 7–8 for the WD group.

The gene expression analysis of markers for sweet sensing receptors in the CV showed an upregulation to 1.29 ± 0.02 (*p* < 0.05) of *Slc2a1*, encoding for Glut-1, in mice that consumed SSB compared to water in the WD group, but not for the other marker genes, *Tas1r2* and *Tas1r3*, as shown in Figure 6A–C. Concerning the bitter taste receptors, shown in Figure 6D–G, Tas2r124 showed one of the highest gene regulations in the microarray, however the upregulation did not reach the level of statistical significance in PCR analysis, probably due to the higher variance in the gene expression in the SSB/WD group. Moreover, the upregulation of *Tas2r104* in the microarray could only be partly confirmed, with the gene expression tending (fold change 2.14 ± 0.54, *p* < 0.1) to be higher in the SSB/WD group than in the corresponding water control. However, the gene expression of *Tas2r106* was 1.63 ± 0.36 times higher in mice that received SSB over water in the chow group (*p* < 0.05). In addition, the *Tas2r130* gene expression tended to be upregulated to a fold change of 2.94 ± 0.85 (*p* < 0.06) in the SSB/WD group compared to the water-fed group.

## 4. Discussion

In the present study, we investigated whether the long-term consumption of a SSB has a stronger impact on taste function when administered in combination with a prototypical WD compared to regular chow and whether those changes are associated with cardiometabolic markers. For this purpose, CD1 mice received a sugar-sweetened, caffeinated soft drink in combination with regular chow or the prototypical WD for a total of 24 weeks, which represents approximately one quarter of the lifetime of a mouse. As expected, the overall energy intake was higher in mice consuming the WD compared to chow. In addition, we expected that the high sugar intake in the SSB groups would contribute to an increased body weight gain and higher blood glucose concentrations as well. Even though a higher fluid intake was noted in the SSB-fed group, the mice compensated for the additional calories from the sugar-sweetened soft drink and consumed less of the respective solid food. This effect was seen for both the WD and regular chow group. A similar compensatory mechanism has already been described for mice, including the CD1 strain, that consumed less chow when given a sucrose solution [27] or for C57/Bl6 mice that received a sugar-containing soft drink [28]. The SSB used in the present study contained glucose-fructose syrup, which is likely to lead to similar compensatory effects as described for a sucrose solution [27]. In addition, it cannot be completely ruled out that the caffeine in the sugar-sweetened soft drink led to a decreased food intake by suppressing appetite. A satiating effect of caffeine has been described before [29], which has been attributed to caffeine’s antagonistic activity on the adenosine receptor A1R in the paraventricular nucleus [30]. However, high caffeine doses of >10 µg injected intracerebroventricularly were required for an appetite-suppressing effect of caffeine in mouse hypothalamic neurons [30]. In the used SSB, a concentration of 80 mg/L of caffeine is described in the ingredients’ list, and with a mean intake of 16.9 mL in both SSB groups per mouse per day, it cannot be said for sure that the required concentration was reached in the hypothalamic region after oral intake of the SSB. 

Since mice of the outbred model CD1 used in the present study rapidly gained weight on a high-fat diet with 60% fat content in previous studies [31,32], we expected that the mice in the present study would also gain weight based on the WD compared to chow, as the WD provides more metabolizable calories from fat. Despite the overall higher energy intake in the WD groups compared to the chow groups, there was no difference in the body weight, lean/fat mass ratio, or the waist circumference between the feeding groups, although there was a trend for a higher body weight after SSB consumption compared to water in the chow group. A higher body temperature of mice consuming the WD pointed to a higher degree of thermogenesis. Since no difference in the amount of BAT was detected, a diet-induced thermogenesis based on the increased amount of food intake is assumed. A thermogenic effect of caffeine can be excluded though, since there was also no difference in body temperature or the BAT between SSB and water groups in both types of diet. To summarize, increased diet-induced thermogenesis may have contributed to the lack of body weight gain of the CD1 mice on the WD, but further markers would be required to prove this assumption. Since the study design was not intended to investigate thermogenesis, no further markers in this direction were analyzed that could give more insights at this stage.

In addition, the lipid profile did not differ between the feeding groups at the end of the experiment, even though more fat was consumed with the WD. As expected, based on the previously described association between the intake of sugar-sweetened beverages and risk for type II diabetes [33], the long-term SSB consumption led to increased fasting glucose levels in the chow group and correlated with the waist circumference, a direct marker for central obesity, which is in accordance with previous studies [34,35,36]. The surrogate marker for insulin sensitivity, QUICKI, indicated that the consumption of the WD, but not the SSB, induced insulin resistance. A negative impact of Western-type diets on insulin sensitivity in mice has been described before [37], however, in contrast to our expectation, the high consumption of glucose-fructose syrup in the SSB group did not aggravate this effect. The reason for this may be the already higher fasting plasma glucose concentration in the WD/water group. Thus, a stronger effect of the WD is concluded and the additional effect of the SSB on fasting glucose concentrations in the WD was not strong enough to reach the level of statistical significance. However, AUC in response to the glucose challenge did not differ between the groups. As we did not determine insulin levels during the oGTT, it remains unclear whether insulin needed to maintain glycemia differed between the groups or not. To clarify the impact of the SSB on insulin sensitivity, further studies using an euglycemic clamp are needed.

Overall, the data of body weight and composition, blood pressure, heart rate, and lipid and glucose metabolism after SSB consumption for 24 weeks in combination with a WD or chow do not hint towards metabolic impairment, which is confirmed by the continuous metabolic score. However, the overall higher energy intake in mice receiving the WD argues for differences in food preference. Since a preference for fat [38] and sugar [27] in CD1 mice has been previously suggested as an innate behavior and the WD contained more fat and sugar than the standard maintenance chow, it seems likely that mice preferred WD and therefore ate more. Moreover, there was a higher fluid intake in the SSB groups, which also hints to a preference for the soft drink. Taste is one of the most important physiological factors regulating food intake [8]. However, taste function can be impaired in humans and rodents with obesity [11]. Several studies demonstrate that obesity is associated with a disturbed taste function on both the morphological and molecular level, which was recently summarized by Harnischfeger & Dando [39]. In addition, one recent study found that exposure to a high-fat diet for six to eight weeks had an impact on taste markers in mice, which is also independent of body weight gain [14]. In addition, a study in *Drosophila melanogaster* showed that a diet high in sugar led to a disturbed sweet taste function without the necessity of obesity [15]. Thus, although the CD1 mice in the present study did not show significant signs of the metabolic syndrome after the long-term SSB intake in chow or WD groups, we were still interested in potential changes in taste markers based on the long-term intake of sugar. We hypothesized that the oral intake of SSB will have a stronger impact on taste when applied in combination with a typical, energy-dense WD. As markers for taste, the number and size of the fungiform papillae, as well as expression levels of genes associated with taste in the circumvallate papillae, were analyzed. The data show that the type of beverage (SSB vs. water) had an impact on the number of fungiform papillae but was independent of the type of diet. The overall chemosensory surface of the fungiform papillae, calculated from the number and size (area) of the fungiform papillae anterior of the intermolar eminence, was significantly reduced after long-term SSB intake in the WD group. A similar trend was seen for the chow groups, which did not reach the level of significance. This leads to the conclusion that the long-term exposure to high amounts of sugar had a stronger impact on the chemosensory surface of the fungiform papillae when applied in combination with the prototypical WD with a higher fat and lower protein content, independent of an increased body weight. A high-fat diet was previously shown to influence the number of fungiform papillae in other studies as well. For example, Kaufman et al. showed that C57Bl/6 mice had a lower fungiform papillae density after receiving a high-fat diet (60% fat) for eight weeks, which was inversely correlated with the body weight [11]. In contrast, in the present study, neither the consumption of the high-fat containing WD, nor the sugar-sweetened soft drink alone for 24 weeks led to a reduced chemosensory surface in CD1 mice. Since Kaufman et al. [11] demonstrated a strong inverse relation between the fungiform papillae density and increased body weight, the lack of an increased body weight in our study provides an explanation for this discrepancy. However, the results presented here demonstrate that the combination of high fat and high sugar may act synergistically and lead to a reduced chemosensory surface also without the onset of obesity or (associated) cardiometabolic disorders. 

Previous studies demonstrated a reduced expression of several taste cell markers, such as PLCβ2 and α-gustducin, in the circumvallate papillae of C57/Bl6 mice after six to eight weeks of a high-fat diet [11,14]. However, the effect of long-term sugar intake on taste marker expression is less well investigated. Thus, we addressed potential changes on the molecular level of taste markers by analyzing the gene expression of several taste markers using a microarray and qRT-PCR from the circumvallate papillae. No changes were detected in the marker genes for type I, II, and III taste receptor cells in response to long-term SSB consumption in combination with the chow or WD. In accordance with that, the genes *Krt8* and *Kcnq1*, used as markers for all differentiated taste cells, were not differently regulated upon SSB consumption. Although the genes *Sox2* and *Ki67* did not hint to changes in proliferating cells, the cell type IV marker genes *Hes6* and *Shh*, were upregulated after long-term SSB consumption in WD, but not in the chow groups. Type IV cells are also referred to as basal or precursor cells that occur near the base of the taste buds and represent newly-generated taste precursor cells that differentiate into functional taste receptor cells [40]. The expression of both, *Hes6* and *Shh,* is related to the nerve innervation of taste buds [41] and both signaling molecules are required for taste receptor cell differentiation, although the role of Shh is better understood [42]. The increased expression of *Shh* and *Hes6* in CD1 mice that received SSB in combination with a WD can be a sign of new taste bud formation. Since the number of taste buds in the circumvallate papillae was not determined in the present study, an increased number of taste buds cannot be experimentally confirmed at this stage. 

In addition, the expression of selected genes associated with taste and taste signaling in the circumvallate papillae was analyzed with pooled RNA samples per group using a customized microarray. According to the hypothesis that the long-term intake of higher amounts of sugar display stronger effects when applied in combination with a WD, the effects of the SSB groups were normalized to the water groups for each diet. Consistent with the hypothesis, the selected genes revealed to be more upregulated when the sugar-sweetened SSB was applied in combination with a WD. The strongest regulation was observed for genes encoding for certain bitter taste receptors (*Tas2r*), followed by members of the *Tas1r* family, encoding for sweet and umami receptors. Validation of gene expression with qPCR showed that SSB consumption in both dietary intervention groups did not result in significant changes of *Tas1r2* or *Tas1r3* expression in the circumvallate papillae. In contrast, Ahart et al. found a down-regulation of *Tas1r3* in mice after 8 weeks on a high fat diet [14]. However, the effect was associated with body weight gain, which provides an explanation as to why no regulation was seen in our study. Still, we found an upregulation of *Slc1a1*, encoding for the glucose transporter 1, in SSB-fed mice receiving WD compared to their water-fed littermates. Glucose transporters have been suggested as an alternative pathway for sugar-sensing beside the canonical sweet taste receptor [43,44]. The upregulation could be interpreted as a counter-regulation to the reduced chemosensory surface in response to SSB consumption in WD groups, but this remains speculative. In addition, qPCR confirmed a trend for an upregulation of *Tas1r130* and *Tas2r104* in mice receiving SSB compared to water in the WD group. Moreover, *Tas2r106* was upregulated in SSB-fed mice compared to water-fed mice in the chow group but did not reach the level of significance in the WD group. A down-regulation of markers for palatable foods in obese subjects has been described in the literature [39], but the effects of diet or body weight gain on the expression of aversive bitter taste receptors have not been well described at this stage. The strongly regulated bitter taste receptors *Tas2r130*, *Tas2r104*, and *Tas2r106* are targeted by caffeine. Since the applied SSB contained caffeine, the upregulation could be a consequence of the long-term exposure to an agonist of the receptor. The high amount of sugar present in the SSB masks the bitterness of caffeine, which explains why the mice still showed a strong preference for the SSB, despite the presence of aversive bitter-tasting caffeine. Whether this effect is solely an effect on the mRNA level, or if the effect might be a counter-regulation to the lower chemosensory surface with an increased sensitivity of the mice towards bitter compounds, such as caffeine, needs to be investigated in future studies. 

This is also one of the main limitations of the present study, since no actual taste preference experiments to determine taste sensitivity of the mice were carried out and no protein levels of the bitter taste receptors could be determined due to the lack of specific antibodies. In addition, the Western-type diet used in the present study was chosen to be as close as possible to a typical Western human diet. However, we cannot exclude that the differences in the micronutrient composition in the diets may have contributed to the observed effects. Future studies are needed to compare the cheeseburger-based diet to a commercialized high-fat diet. Furthermore, the data cannot be completely transferred to humans, since regulation of food intake in humans is more strongly influenced by psychological and socioeconomic factors, and thus potential physiological compensatory mechanisms in calorie intake may be overruled in humans. Therefore, future translational studies in humans are required to understand the putatively vicious cycle of diet choices and taste (dys-)function.

## 5. Conclusions

The present study shows that in CD1 mice, long-term consumption of SSB reduced the chemosensory surface of the fungiform papillae when applied in combination with a WD without the necessity of an increased body weight, suggesting synergistic effects of a high sugar and high fat diet on taste dysfunction. Moreover, an increased expression of markers for basal cells of the taste buds and bitter taste receptors targeting caffeine in the circumvallate papillae of WD SSB-fed mice could argue for compensatory mechanisms towards the reduced chemosensory surface. The consequences for food intake and preference of the found changes of taste markers need to be investigated in future studies. In addition, the reduction of the chemosensory surface could be a forerunner of obesity and future studies should investigate whether taste dysfunction aggravates after manifestation of obesity.

## Data Availability

Data are available on request from the corresponding author.

## Supplementary Materials

The following supporting information can be downloaded at: https://www.mdpi.com/article/10.3390/nu14030594/s1, Figure S1: Mean plasma concentrations of (a) high density lipoprotein (HDL), (b) low density lipoprotein (LDL), (c) total cholesterol, and (d) triacylglycerols (TAG) from mice that received either a standard diet (chow) or Western-type diet (WD) with water (blue bars) or a SSB (brown bars) as drink for 24 weeks. Figure S2: Systolic (a) and diastolic blood pressure (b) [both displayed in mm Hg], and heart rate [beats per minute, bpm] (c) of mice receiving either a standard diet (chow) or Western-type diet (WD) with water (blue bars) or a SSB (brown bars) as drink. Figure S3: Continuous metabolic syndrome score calculated from the z-scores of fasting glucose, waist circumference, systolic blood pressure, TAG, and HDL of mice receiving either a standard diet (chow) or Western-type diet (WD) with water (blue bars) or a SSB (brown bars) as drink. Table S1: List of primers used in the present study.
