# Supplementary material for: Long-Term Consumption of a Sugar-Sweetened Soft Drink in Combination with a Western-Type Diet Is Associated with Morphological and Molecular Changes of Taste Markers Independent of Body Weight Development in Mice
Source: Nutrients. 2022 Jan 29;14(3):594. doi: 10.3390/nu14030594 (PMC8837962; doi:10.3390/nu14030594)
Supplement: Supplementary file 1 [file nutrients-14-00594-s001.zip › nutrients-1560220-supplementary.pdf]

Long-term consumption of a sugar-sweetened soft drink in combination with a Western-type diet is associated with morphological and molecular changes of taste markers independent of body weight development in mice

Barbara Lieder [*#1], Jozef Čonka [#2], Agnes T. Reiner [1], Victoria Zabel [1], Dominik Ameur [3], Mark M. Somoza[3,4,5], Katarína Šebeková[2], Peter Celec[2], Veronika Somoza[1,4,6]

# equal contribution
*Correspondence: Barbara.Lieder@univie.ac.at

Supplemental Material

Table S1. List of primers used in the present study

Gene	Sequence<br>Forward Primer, Reverse Primer	Product size [bp]
18S	CGGCTACCACATCCAAGGAA<br>GCTGGAATTACCGCGGCT	187
ACTB	CCCTGTGCTGCTCACC<br>GCACGATTTCCCTCTCAG	328
Car4	TACGTGGCCCCCTCTACTG<br>GCTGATTCTCCTTACAGGCTCC	115
Entpd2	TGCGCCTAAACCTGAC<br>CCCAGCCATACTTGATGAAGTT	183
GLUT1	CAGTTCGGCTATAACACTGGTG<br>GCCCCCGACAGAGAAGATG	156
GNAT3	GCAACCACCTCCATTGTTCT<br>AGAAGAGCCCACAGTCTTTGAG	286
Hes6	ACCACCTGCTAGAATCCATGC<br>GCACCCGGTTTAGTTCAGC	210
HPRT	GAGAGCGTTGGGCTTACCTC<br>ATCGCTAATCACGACGCTGG	136
KCNQ1	ATGCTCTGTGGTGGGGGGTG<br>CTTCTGCCTCTGCTTCTGCTGG	185
Ki67	ATCATTGACCGCTCCTTTAGGT<br>GCTCGCCTTGATGGTTCCT	104
Krt8	TACATCAACAACCTCCGCCG<br>GCAACTCACGGATCTCCTCTT	188
Shh	AAAGCTGACCCCTTTAGCCTA<br>TTCGGAGTTTCTTGTGATCTTCC	103
Slc1a3	ACTGCTGTCATTGTGGGTACA<br>ATAGACTACAGCGCGCATCC	213
Snap-25	CAACTGGAACGCATTGAGGAA<br>GGCCACTACTCCATCCTGATTAT	177
Sox2	GCGGAGTGGAAACTTTTGTCC	157

	CGGGAAGCGTGTACTTATCCTT	
Tas1r2	AAGCATCGCCTCCTACTCC<br>GGCTGGCAACTCTTAGAACAC	114
Tas1r3	GAAGCATCCAGATGACTTCA<br>GGGAACAGAAGGACACTGAG	283
Tas2r104	CAAAGGTTTTCCTTCTGACTATGCT<br>CAAACGCTCAGATGGTTAATAATTACC	91
Tas2r106	TATATGGTTTGCCACCAGCCT<br>AGAGGTAAACCTGCCAGGAAA	215
Tas2r124	CAATTCTAGAGGAGATAGAGACCCTAGT<br>G<br>AGCAAGAGGAAGGAGACCAAAA	78
Tas2r130	TGAAAGCCATGAAAGCAGTAATTT<br>CTGGAGGTGGCTATGAGAAAGG	79

Figure S1

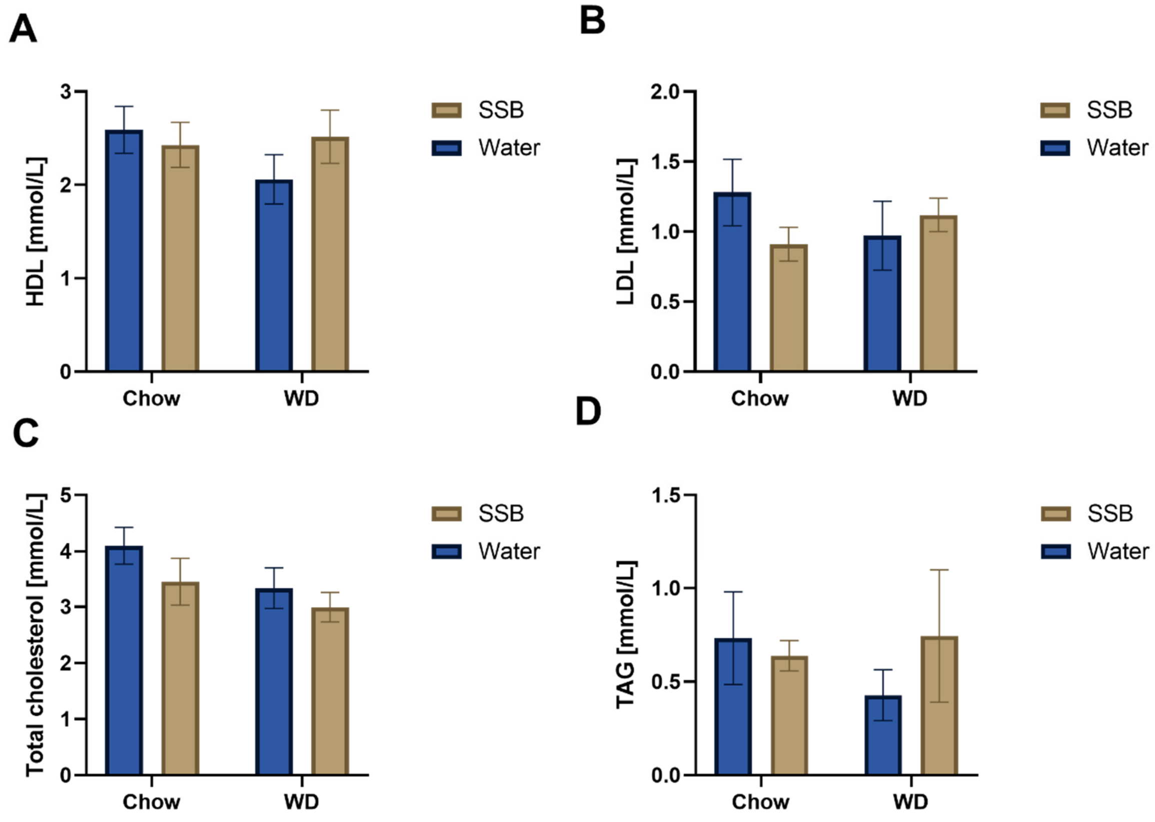

Figure S1. Mean plasma concentrations of **(a)** high density lipoprotein (HDL), **(b)** low density lipoprotein (LDL), **(c)** total cholesterol, and **(d)** triacylglycerols (TAG) from mice that received either a standard diet (chow) or Western-type diet (WD) with water (blue bars) or a SSB (brown bars) as drink for 24 weeks. Statistical significance was tested using two-way ANOVA with Holm-Sidak post hoc test and assumed at $p<0.05$.

Figure S2

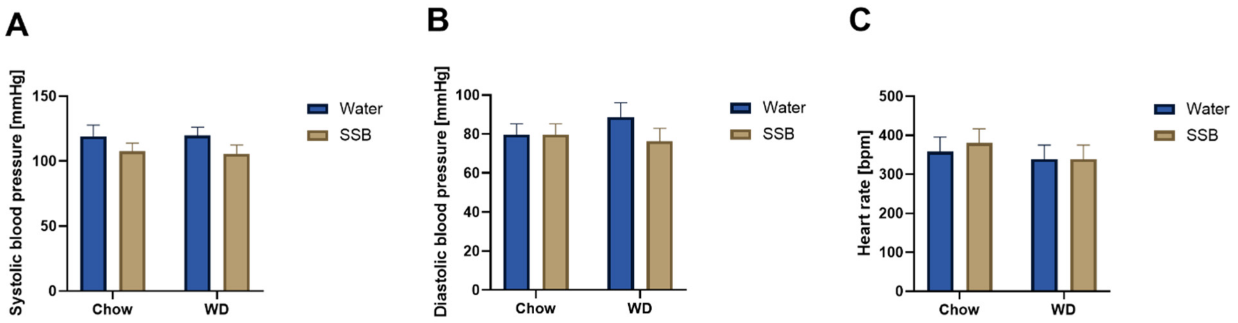

Figure S2. Systolic **(a)** and diastolic blood pressure **(b)** [both displayed in mm Hg], and heart rate [beats per minute, bpm] **(c)** of mice receiving either a standard diet (chow) or Western-type diet (WD) with water (blue bars) or a SSB (brown bars) as drink. All data are shown as mean ± SEM. Statistical significance was tested using two-way ANOVA with Holm-Sidak post hoc test and assumed at $p<0.05$.

Figure S3

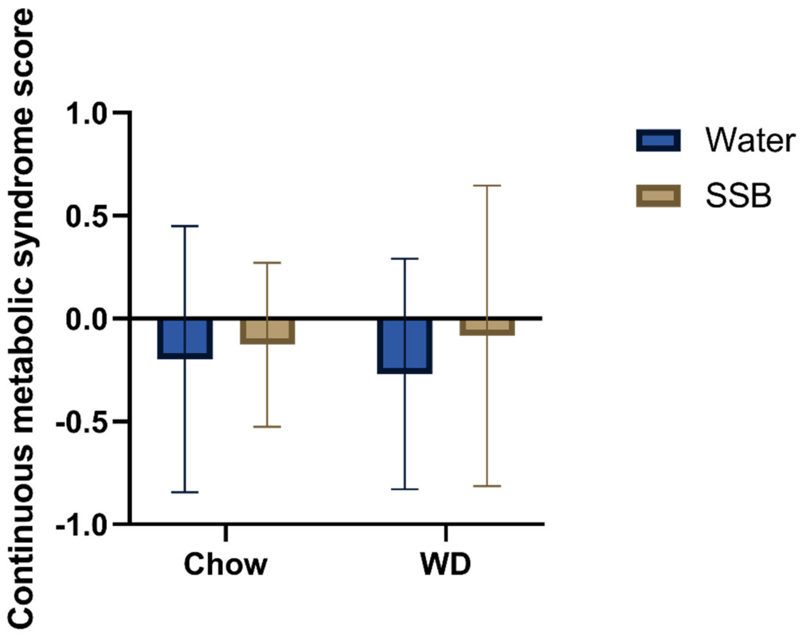

Figure S3. Continuous metabolic syndrome score calculated from the z-scores of fasting glucose, waist circumference, systolic blood pressure, TAG, and HDL of mice receiving either a standard diet (chow) or Western-type diet (WD) with water (blue bars) or a SSB (brown bars) as drink. All data are shown as mean ± SEM. Statistical significance was tested using two-way ANOVA with Holm-Sidak post hoc test and assumed at $p<0.05$.
